# An adult patient with Nijmegen Breakage Syndrome and Hodgkin's Lymphoma

**DOI:** 10.1186/2052-1839-14-2

**Published:** 2014-01-16

**Authors:** Katharina Engel, Martina Rudelius, Felix G Meinel, Christian Peschel, Ulrich Keller

**Affiliations:** 1 Department of Medicine III, Technische Universität München, Ismaninger Strasse 22, 81675 Munich, Germany; 2 Institute of Pathology, Technische Universität München, Munich, Germany; 3 Institute for Clinical Radiology, Ludwig-Maximilians-Universität, Munich, Germany

**Keywords:** Nijmegen breakage syndrome, DNA repair disorder, Hodgkin lymphoma, ABVD chemotherapy

## Abstract

**Background:**

Nijmegen Breakage Syndrome (NBS) is a rare autosomal recessive DNA repair disorder characterized by immune deficiency, microcephaly, mental retardation and a disposition for the development of hematological malignancies. So far, mostly pediatric patients have been described, since the underlying condition is often fatal before adulthood. Many patients diagnosed with Hodgkin lymphoma (HL) due to this DNA repair defect receive reduced treatment followed by early progression and fatal outcome.

**Case presentation:**

We describe here a 26-year old male caucasian patient with NBS who presented with multi organ failure due to HL. Immediate intensive chemotherapy lead to complete remission and reversed organ failure.

**Conclusion:**

We show that application of standard chemotherapy can lead to long-term disease free survival in patients with a DNA repair disorder. Furthermore, we describe here, to the best of our knowledge, the first adult patient with NBS and HL.

## Background

Nijmegen Breakage Syndrome (NBS) is a rare autosomal recessive DNA repair disorder [1] characterized by a 5 bp deletion in the *NBS1* gene on chromosome 8q21, which leads to a truncated and insufficiently functioning protein Nibrin [2]. By mediating the phospho ATM-driven assembly of BRCA, MRE11 and Rad50 on double strand breaks, nibrin carries a central role in the cellular response to DNA damage [3]. Homozygous state of the above mentioned mutation causes immune deficiency, microcephaly, mental retardation and a disposition for the development of leukemias as well as Hodgkin and Non-Hodgkin lymphomas (HL; NHL) [4-6]. To the best of our knowledge we here describe the first adult patient with NBS and HL.

## Case presentation

### History

A 23 year old male was referred to our intensive care unit from an outside hospital with hepatic and renal failure (serum bilirubin 41 mg/dl, International Normalized Ratio (INR) 1.7, Blood Urea Nitrogen (BUN) 108 mg/dl, serum creatinin 5.3 mg/dl).

**Figure 1:**
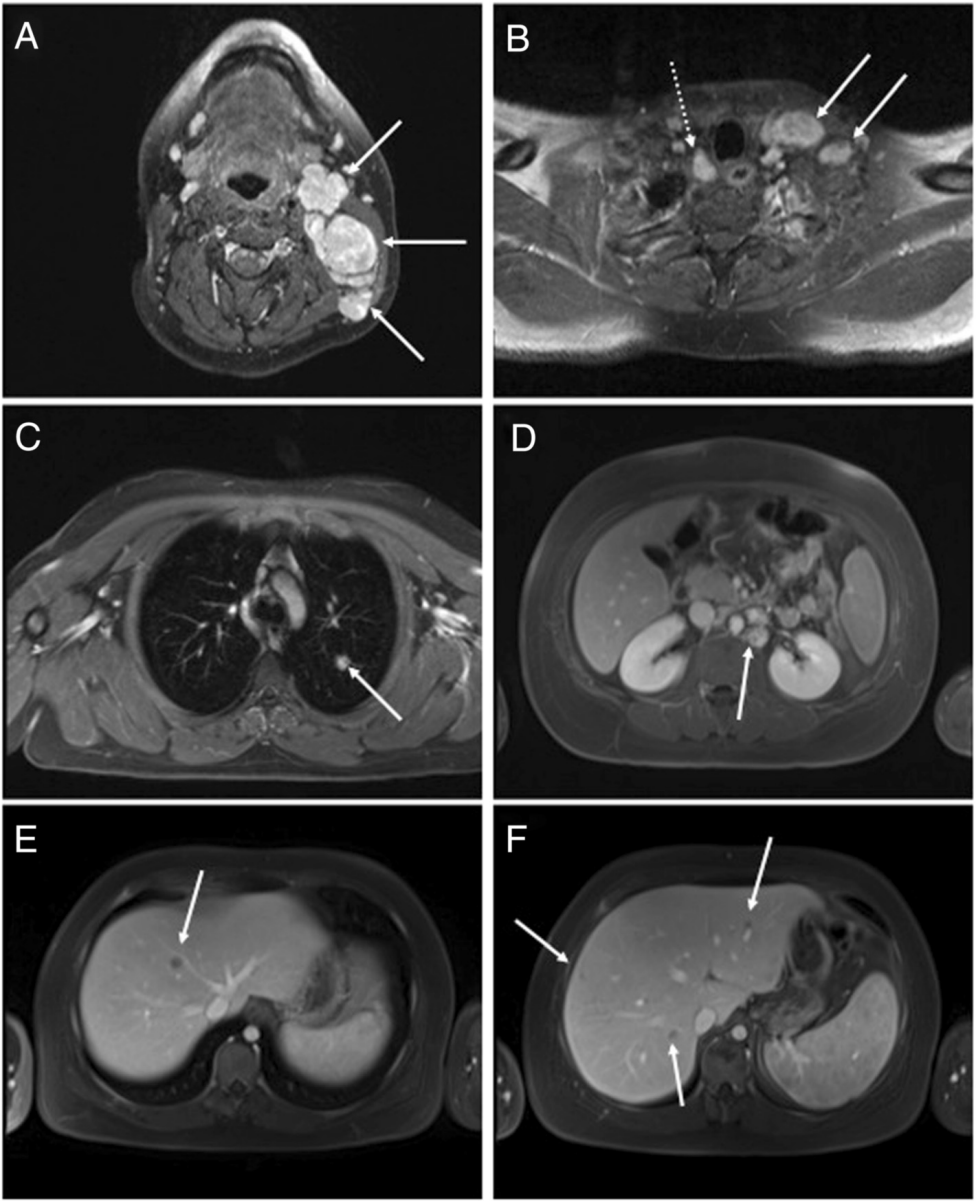
**Initial radiologic staging confirming lymphoma Ann Arbor stage IV. (A)** T1 weighted images after i.v. contrast administration demonstrate left cervical lymphadenopathy (arrows). **(B)** Pathological enlargement and contrast uptake is also noted in left supraclavicular (arrows) and right paratracheal (dotted arrow) lymph nodes. Delayed phase VIBE sequence demonstrates a lung nodule **(C)** and left paraaortic lymphadenopathy **(D)**. Multiple hypointense liver lesions are noted in the VIBE sequence acquired in portal venous phase **(E + F)**. These findings are consistent with HL with nodal, hepatic and pulmonary involvement.

Three weeks earlier the patient had presented to an outside hospital with increasing fatigue and cervical swelling over several weeks. Biopsy of the cervical lymph nodes had established the diagnosis of nodular sclerosing classical HL. Staging was done by whole body MRI scan (Figure 1) and revealed a large cervical lymph node bulk (8 × 3.3 cm) on the left side and slightly enlarged cervical, paratracheal and paraaortal lymph nodes on the right side. Moreover, there were two small masses in the left lung (13 mm and 7 mm in diameter, respectively) as well as multiple hepatic nodules. Further laboratoy work-up was notable for increased Erythrocyte Sedimentation Rate (ESR) of 96 mm/h, and immunodeficiency (lymphocytopenia, T helper cell and B cell count significantly decreased, IgG4 deficiency). Histological examination of the bone marrow showed no infiltration by HL.

The patient was also known to suffer from NBS. He was diagnosed at birth and registered in the NBS Registry as patient No. 45. In accordance with this diagnosis he displayed mild mental retardation (he had been working as an auxiliary cook up to this point), microcephaly, characteristic facial features as well as an immune defect. He was known to suffer from selective IgG4 deficiency and his fibroblasts and lymphocytes had been found to be hypersensitive to induction of DNA breaks (The International Nijmegen Breakage Study Group [7]). Up until this young adult age he had no history of severe infections.

During the initial evaluation the patient was withdrawn from further medical care by his parents. When he developed B symptoms and his condition rapidly deteriorated he was readmitted and transferred to our hospital. The patient measured 1,52 m with a weight of around 75 kg; he presented awake and oriented, but severely icteric, anuric and edematous. On admission, a little over two weeks after the diagnosis had been established, he was in acute renal and hepatic failure. Treatment with Prednisone 100 mg i.v. daily had been started.

### Clinical course

**Figure 2:**
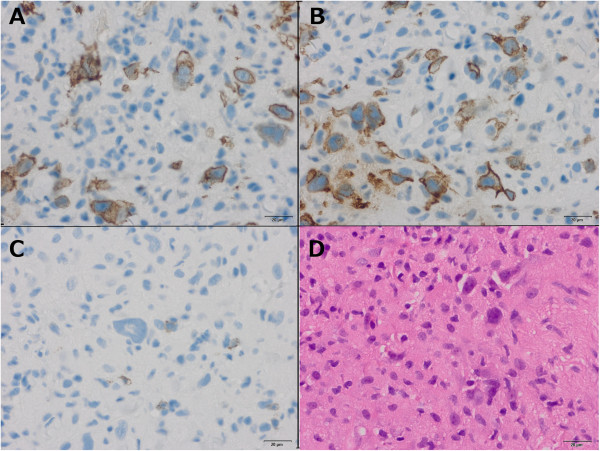
**Liver Biopsy documenting hepatic involvement of Hodgkin’s lymphoma. (A)** Histology of classical HL with lacunar cells and mixed inflammatory background. Hodgkin cells express a characteristic immunophenotype with strong positivity for CD30 **(B)** and weak expression of CD20 **(C)**. CD15 staining **(A)** highlights few histocytes. **(D)** Histology of classical HL with lacunar cells and mixed inflammatory background.

Ultrasound-guided biopsy of liver nodules was performed immediately and showed multiple small nodular infiltrates of classical Hodgkins lymphoma (Figure 2). Therefore, diagnostic stage had to be corrected to IVB and systemic chemotherapy similar to CHOP schedule was started, however adapted to poor renal and hepatic function. He received reduced CHOP-like chemotherapy including cyclophosphamide 375 mg/qm (50%), doxorubicin 12.5 mg/qm (25%) and vincristin 2 mg abs. on day 3 of the hospital stay.

Citrate hemodialysis was initiated immediately and continued for the following four weeks. During this time renal function started to improve. Additionally, after application of the first cycle of chemotherapy, albumin dialysis was applied several times until hepatic function started to recover.

While organ functions were improving following application of chemotherapy, the patient developed aspergillus fumigatus and candida albicans pneumonia, confirmed by culture of bronchioalveolar fluid, and had to be ventilated for ten days. White blood counts were stimulated using granulocyte colony stimulating factor (G-CSF) starting on day 4 after chemotherapy application. Twenty-one days after the first chemotherapy, the patient received the second cycle, this time an attenuated ABVD schedule (adriamycin d1 75% and d15 100%, bleomycin d1 25% and d15 50%, vinblastin d1 75% and d15 100%, dacarbazin d1 50% and d15 75%). During the second cycle he again became septic from Candida urinary tract infection but recovered in due time with caspofungin treatment and G-CSF support to receive the next dose of chemotherapy on day 15. Overall, we applied four cycles of chemotherapy, three of them ABVD d1 and d15, and were able to escalate the doses of adriamycin, bleomycin and vinblastin to 100%, and of dacarbazin to 75% of the regular adult schedule. In detail, the following doses (given as total or per m^2^ body surface area) were applied. Cycle 1: cyclophosphamide 375 mg/m^2^, doxorubicin 12,5 mg/m^2^, vincristin 2 mg total dose; cyclophosphamide was reduced to limit hematotoxicity considering renal failure; doxorubicin dose was adjusted to poor liver function. Cycle 2 (3 weeks later), d1: doxorubicin 19 mg, bleomycin 2,5 mg, vinblastin 4,4 mg, dacarbazin 194 mg, all per m^2^. doxorubicin was still applied in a lower dose to account for hepatic dysfunction, bleomycin was thought to be particularly toxic since it induces DNA breaks, especially in kidney failure. Since the patient was still recovering from severe sepsis also vinblastin and dacarbazin were reduced to limit the risk of neutropenic infection. Pegylated G-CSF was given d4 and d19. Cycle 2, d15: doxorubicin 25 mg, bleomycin 5 mg, vinblastin 6 mg, dacarbazin 281 mg, all per m^2^. Cycle 3 and cycle 4, d1 and d15: doxorubicin 25 mg, bleomycin 10 mg, vinblastin 6 mg, dacarbazin 281 mg, all per m^2^.

However, the patient was affected with severe intensive care polyneuropathy leading to lower extremities paresthesias and paralysis. The situation was further complicated by grade IV anal ulcer and eventually a depressive episode. The resulting refusal to co-operate prevented mobilisation as well as implementation of supportive measures and staging procedures. Therefore, and in consideration of the dramatic clinical response already obtained it was decided together with his family to terminate chemotherapy. After fourteen days on an inpatient psychiatric ward undergoing intensive psychotherapy the patient was referred to a rehabilitation facility for the following eight weeks and then discharged home.

Follow-up MRI was performed at 3, 6, 12 and 18 months after initial diagnosis and continued to show complete remission. At 20 months after the initial diagnosis, the patient has now regained full mobility, does not exhibit any clinical sequelae of the disease and has started to work again as an auxiliary cook. His mental condition has returned to baseline without pharmacologic intervention.

## Conclusions

NBS is an autosomal recessive DNA repair disorder similar to Ataxia Teleangiectasia (AT), which predisposes for the development of HL and NHL. So far, 25 pediatric, adolescent and young adult patients with DNA repair disorder – Ataxia Teleangiactasia or Nijmegen Breakage Syndrome - and HL have been identified [8,9]. The median age at time of diagnosis was 9.4 years with two ATM patients being of adult age (20 and 25 years). One NBS patient with HL described in the literature was six years old and died 12 months after diagnosis [8]. The other, a five-year old, however, reached sustained remission after intensive chemotherapy [10]. To the best of our knowledge we here describe the first adult patient with NBS and HL.

Although the pathogenetic basis of both conditions resemble each other, NBS and AT display distinct clinical features. NBS, first described in 1981 [11], is characterized by microcephaly, typical facies, immunodeficiency, and chromosomal instability [1]. It lacks the typical cerebellar ataxia that lends its name to AT, but shares the tendency to develop lymphoid malignancies [6,12].

Our patient was affected with microcephaly, reduced intelligence and the typical craniofacial features but never suffered from severe infectious complications despite the lymphocytopenia, IgG4 deficiency and chromosomal instability. When sequencing of the *NBS1* gene was performed at the time of diagnosis no known mutations were identified. Parental genotyping was declined. The patient was thus included into the Nijmegen Breakage Registry based on clinical signs and symptoms, i.e. microcephaly, immunodeficiency and chromosomal instability. Among the cases published within the first report of the Nijmegen Breakage Registry in 2000 [7], in nine out of 55 (16%) patients no mutation was identified and 37 children (67%) presented without a family history.

The overall mild phenotype might explain the late onset of malignant disease: with an age of 23 years he is the second oldest patient with HL and DNA repair disorder described so far. The most common *NBS1* mutation (c.675del5) is hypomorphic [13] and indirectly leads to a N terminally truncated, alternatively translated NBS protein (p70), the level of which determines damage sensitivity in lymphoid cells. Likewise, our patient’s genetic aberration might give rise to a particularly high level of functioning truncated Nibrin protein. However, the genotype-phenotype correlation is unsatisfactory [7] and our patient’s mild phenotype in childhood and adolescence was probably more predictive for treatment tolerance and success than the genetic background could have been.

Although the majority of patients with DNA repair disorder and HL are diagnosed in a far advanced stage of the malignancy [14] the presence of multi organ failure in this clinical setting has not been reported so far. While liver failure was clearly due to HL infiltration, the origin of renal failure could have been a consequence of either sepsis or high cell turn-over but was certainly aggravated by hypovolemia (high fever without adequat fluid substitution) and hepato-renal mechanisms. Tumor infiltration of the kidneys appears unlikely as this was not identified on magnetic resonance scan. Patients with stage IV disease at diagnosis (HL or NHL or other malignancies accompanying this disorder) never survived more than half a year [14]. In our case, the early and rather aggressive initial treatment (anthracyclin, vinca alkaloid and alkylating agent) completely restored organ function by leading to fast and sustained remission of HL. However, the patient described here suffered from two episodes of severe infection, both from fungi rather than bacteria. It has been described that the incidence of fungal infections is higher in NBS patients due to reduced cellular and humoral immunity [15]. In our patient, who had never suffered from infectious complications before, chemotherapy-induced neutropenia might have tipped him over the edge to systemic fungal infection.

Although the patient might have to face secondary malignancy from treatment within the next years, the hitherto clinical course provides strong support for an initial standard ABVD treatment strategy in patients with underlying DNA repair disorder. Importantly, additional iatrogenic DNA damage should be avoided using clinical examination as well as MRT or PET-MRT instead of CT for imaging.

In summary, this is – to the best of our knowledge - the first adult patient with NBS and HL described so far. Despite presenting with multiple organ failure and stage IV disease, he achieved sustained remission with early standard chemotherapy.

## Consent

Written informed consent was obtained from the patient for publication of this case report and any accompanying images. At the time of publication the patient himself was able to evaluate his consent despite his mild mental retardation. However, during the course of his disease, he was legally represented by his parents. Therefore, consent for publication of this work was also obtained from them. A copy of the written consent is available for review by the Editor of this journal.

## Abbreviations

HL: Hodgkins lymphoma; NBS: Nijmegen breakage syndrome; NHL: Non-hodgkins lymphoma; INR: International normalized ratio; BUN: Blood urea nitrogen; ESR: Erythrocate sedimentation rate; G-CSF: Granulocyte colony stimulating factor; AT: Ataxia teleangiectasia.

## Competing interests

The authors declare that they have no competing interests.

## Authors’ contributions

KE wrote the paper and analysed the data. MR contributed the histologic and FM the radiologic data. CP and UK revised the manuscript critically. All authors read and approved the final manuscript.

## Pre-publication history

The pre-publication history for this paper can be accessed here:

http://www.biomedcentral.com/2052-1839/14/2/prepub
